# Synthesis of New Furothiazolo Pyrimido Quinazolinones from Visnagenone or Khellinone and Antimicrobial Activity

**DOI:** 10.3390/molecules23112793

**Published:** 2018-10-27

**Authors:** Ameen Ali Abu-Hashem

**Affiliations:** 1 Photochemistry Department (Heterocyclic Unit), National Research Centre, Dokki, Giza1 2622, Egypt; aminaliabuhashem@yahoo.com; Tel.: +2-01225211700 or +966-591363915; Fax: + 202-33370931; 2 Chemistry Department, Faculty of Science, Jazan University, 2097 Jazan, Saudi Arabia

**Keywords:** visnagenone, khellinone, quinazolinone, pyrimidine, thiazole, pyrazole, furane, antimicrobial activity

## Abstract

Substituted-6-methyl-1-thioxo-1,2-dihydro-3*H*-furo[3,2-g]pyrimido[1,6-a]quinazolin-3-ones (**5a**,**b**) were synthesized from condensation of visnagenone (**2a**) or khellinone (**2b**) with 6-amino-thiouracil (**3**) in dimethylformamide or refluxing of (**4a**) or (**4b**) in dimethylformamide. Hence, compounds (**5a**,**b**) were used as the starting materials for preparing many new heterocyclic compounds such as; furo[3,2-g]pyrimido[1,6-a]quinazoline (**6a**,**b**), furo[3,2-g]thiazolo[2′,3′:2,3]pyrimido[1,6-a]quinazolinone (**7a**,**b**), substituted-benzylidene-furo[3,2-g]thiazolo[2′,3′:2,3]pyrimido[1,6-a]quinazoline-3,5-dione (**8a**–**f**), 3-oxo-furo[3,2-g]pyrimido[1,6-a]quinazoline-pentane-2,4-dione (**9a**,**b**), 1-(pyrazole)-furo[3,2-g]pyrimido[1,6-a]quinazolinone (**10a**,**b**), 2-(oxo or thioxo)-pyrimidine-furo[3,2-g]pyrimido[1,6-a]quinazolinone (**11a**–**d**), 1-(methylthio)-furo[3,2-g]pyrimido[1,6-a]quinazolinone (**12a**,**b**), 1-(methyl-sulfonyl)-furo[3,2-g]pyrimido[1,6-a]quinazolinone (**13a**,**b**) and 6-methyl-1-((piperazine) or morpholino)-3*H*-furo[3,2-g]pyrimido[1,6-a]quinazolin-3-one (**14a**–**d**). The structures of the prepared compounds were elucidated on the basis of spectral data (IR, ^1^H-NMR, ^13^C-NMR, MS) and elemental analysis. Antimicrobial activity was evaluated for the synthesized compounds against Gram-positive, Gram-negative bacteria and fungi. The new compounds, furothiazolo pyrimido quinazolines **8a**–**f** and **11a**–**d** displayed results excellent for growth inhibition of bacteria and fungi.

## 1. Introduction

**Figure 1:**
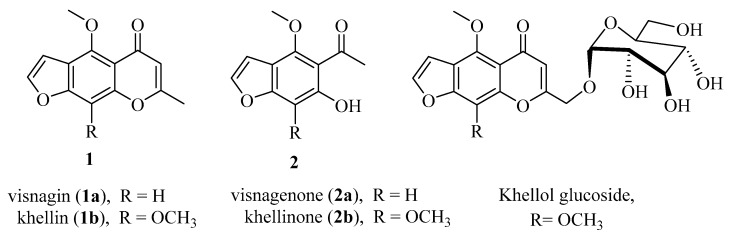
Chemical structure of furochromone derivatives.

Physicians confirm the usefulness of adding many naturally occurring drugs. Furochromones (visnagin and khellin) compounds are natural products extracted from the plant of *Ammi visnaga Lam*. and visnagin and khellin are used as therapy for kidney, bladder stones, diuretic infusions [1,2] and are considered essential components of many drugs [3]. As well, furochromone derivatives are reported as anti-atherosclerotic, antineoplastic, anti-gastric, anti-anaphylactic and are used in the treatment of urolithiasis, hypertriglyceridemia [4,5] and vitiligo [6]. Also, the visnaginone derivatives were synthesized using different procedures and elucidated efficient antimicrobial activities [7,8]. Furthermore, furochromones have been used to treat pain in the renal colic [9] and possesses coronary vasodilating activity [10,11]. Moreover, khellin and visnagin have been used in the photo-chemotherapeutic treatment of vitiligo and psoriasis [12], photoreaction with DNA [13] and Khellin displayed important epidermal growth factor receptor (EGFR) inhibitory activity [14]. Benzofurans and furochromones [15,16,17,18] are very exciting heterocycles, which are omnipresent in nature and display a wide range of pharmacological activities. Recently, furochromones and benzofurans derivatives are used as in antiviral and anticancer activities [19,20]. When fused of furochromones with pyrimidine, quinoxaline and pyrazole derivatives showed anti-inflammatory and analgesic activities [21], the cytotoxic activity [22] and used in the protection of DNA [23]. Additionally, some of the moieties heterocyclic such as, chalcones [24], thiazolidinones [25], Mannich bases [26], sulfonamides [27], and isoxazole [28], too, showed several of biological activity with benzofuran derivatives (visnagenone, khellinone) (Figure 1).

An extension of our work on the preparation of novel heterocyclic compounds resultant from the naturally occurring visnagin and khellin [21,22,23,29], we synthesized and described several derivatives which contained a benzofuran moiety (visnaginone, khellinone) incorporated with thiazole, pyrimidine, pyrazole, and quinazolinone derivatives. The antimicrobial activity of the prepared compounds were evaluated.

## 2. Results and Discussion

### 2.1. Synthesis

**Scheme 1:**
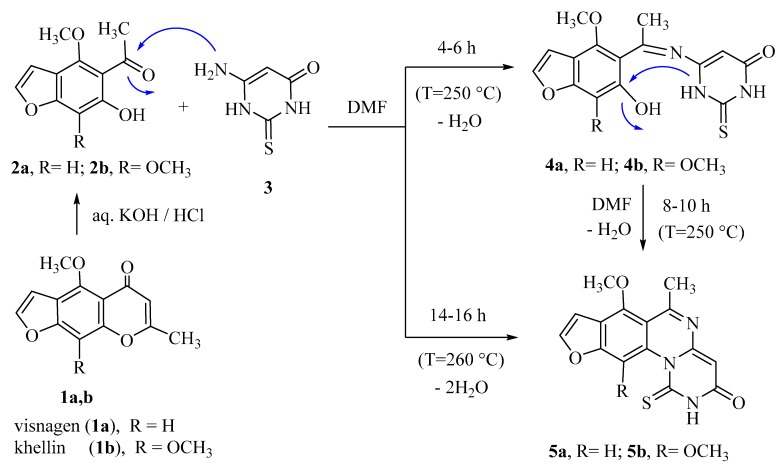
Synthesis of furopyrimido quinazolinones from visnagenone and khellinone derivatives.

In the present work, the natural furochromones (visnagin **1a** or khellin **1b**) are highly sensitive to alkali. Hence, aqueous alkaline hydrolysis of **1a** and **1b** using potassium hydroxide with heating and stirring at 40–50 °C for 1–2 h lead to form visnagenone **2a** or khellinone **2b**, respectively [22,23]. Moreover, heating under reflux cyclic alfa, beta-unsaturated ketones (visnagenone **2a** or khellinone **2b**) with 6-aminothiouracil (**3**) in dimethylformamide solution [21,22] with stirring for 4–6 h or 14–16 h under (TLC) afforded new compounds respectively, 6-((1-(6-hydroxy-(4-methoxy or 4,7-dimethoxy)-benzofuran-5-yl)ethylidene)amino)-2-thioxo-2,3-dihydropyrimidin-4(1*H*)-one (**4a**,**b**) and (7-methoxy or 7,11-dimethoxy)-6-methyl-1-thioxo-1,2-dihydro-3*H*-furo[3,2-g]pyrimido[1,6-a]quinazolin-3-one (**5a**,**b**) in good yield obtained. Also, another method, stirring under reflux of **4a** or **4b** in dimethylformamide solution for 8–10 h afforded the same products **5a** and **5b**, respectively. The ^1^H-NMR spectrum of compound **4a** showed three singlet broad signal at 10.80, 11.19 and 16.11 ppm corresponding to the three protons of the (2NH) and one (OH) groups, which were D_2_O exchangeable and ^1^H-NMR spectrum of compound **5a** showed one singlet broad signal at 11.20 ppm conforming to the one proton of the one (NH) group (D_2_O exchangeable) and the mass spectra of **4a**, **4b**, **5a** and **5b** showed molecular ion peaks at *m/z* 331 (M^+^, 100%) and 361 (M^+^, 100%), 313 (M^+^, 100%), 343 (M^+^, 100%) respectively (Scheme 1).

**Scheme 2:**
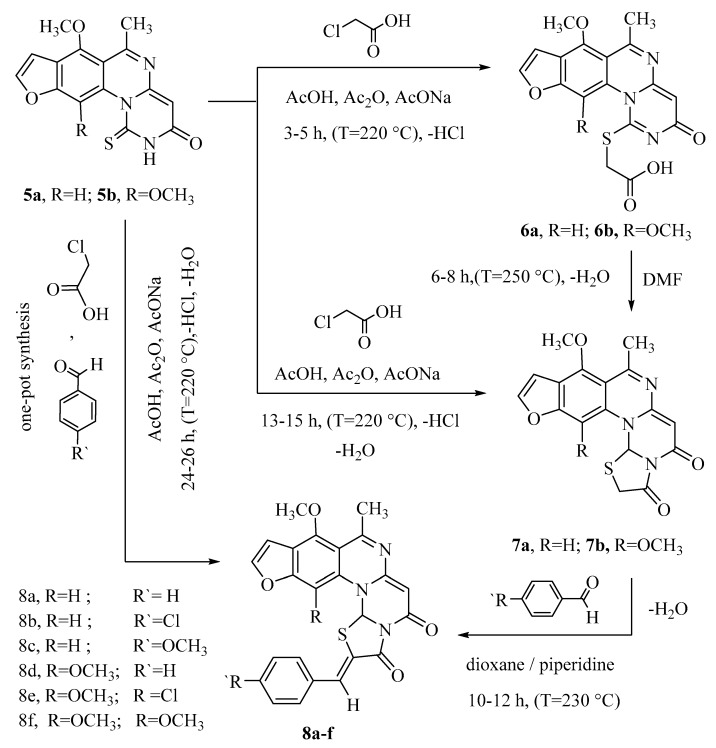
Synthesis of furothiazolopyrimidoquinazolinone derivatives.

In this research, simple and convenient methods to the syntheses of furopyrimido quinazoline, furothiazolo pyrimido quinazolinone and furothiazolo pyrimido quinazolinone benzylidene derivatives [30]. Thus, refluxing of **5a** or **5b** with chloroacetic acid in glacial acetic acid/acetic anhydride and anhydrous sodium acetate for 3–5 h or 13–15 h with control via (TLC) to give new compounds followed by, 2-(((7-methoxy or 7,11-dimethoxy)-6-methyl-3-oxo-3*H*-furo[3,2-g]pyrimido[1,6-a]quinazolin-1-yl)thio)acetic acid (**6a**,**b**) and (9-methoxy or 9,13-dimethoxy)-8-methyl-2-hydro-5*H*,14a*H*-furo[3,2-g]thiazolo[2′,3′:2,3]pyrimido[1,6-a]quinazoline-3,5-dione (**7a**,**b**) in high yields. Moreover, the boiling of **6a** or **6b** in dimethylformamide solution resulted in the same formation of **7a** and **7b**. The IR spectrum of compounds **6a** and **6b** exposed the presence of broad band absorption at 3340–3350 cm^−1^ indicative of one (OH) group and **6a** = 1745, 1684 cm^−1^ and **6b** = 1748, 1681 cm^−1^ of the two carbonyl groups. The ^1^H-NMR spectrum of **6a** showed a singlet broad signal at 13.70 ppm corresponding to the one protons of the one (OH) group, which were D_2_O exchangeable. Also, ^1^H-NMR spectrum of **7b** displayed a five singlet signal at δ 2.33, 3.97, 4.24, 5.62 and 7.39 ppm conforming to the thirteen protons of the methyl, two methoxy, (CH_2_), (CH, thiazole) and (CH, pyrimidine) groups, respectively. As well as using one-pot synthesis as follows: when a ternary mixture of **5a** or **5b**, chloroacetic acid and a proper aldehyde namely; benzaldehyde, 4-chlorobenzaldehyde or 4-methoxybenzaldehyde respectively, was heated under reflux in a mixture of acetic acid and acetic anhydride in the presence of anhydrous sodium acetate afforded the conforming 2-(substituted-benzylidene)-9,(substituted)-methoxy-8-methyl-2-hydro-5*H*,14a*H*-furo[3,2-g]thiazolo[2′,3′:2,3]pyrimido[1,6-a]quinazoline-3,5-dione (**8a**–**f**) in high yields. In another route, we obtained on the same compounds (**8a**–**f**) via refluxing of **7a** or **7b** with appropriate aromatic aldehyde in dioxane solution containing a catalyst amount of piperidine for 10–12 h. ^1^H-NMR spectrum of **8a** showed six singlets at 2.30, 3.91, 5.59, 7.32, 7.85 and 8.05 ppm conforming to the 10 protons of the methyl, methoxy, thiazole proton, pyrimidine proton, phenyl proton and (CH) proton groups, respectively and two doublet signals at 6.75, 7.72 ppm of the two protons (*J* = 2.30 Hz, furan). The structures assignments for compounds were established on their elemental analysis and spectral (IR, ^1^H, ^13^C-NMR, and MS) data are shown in the experimental section (Scheme 2).

**Scheme 3:**
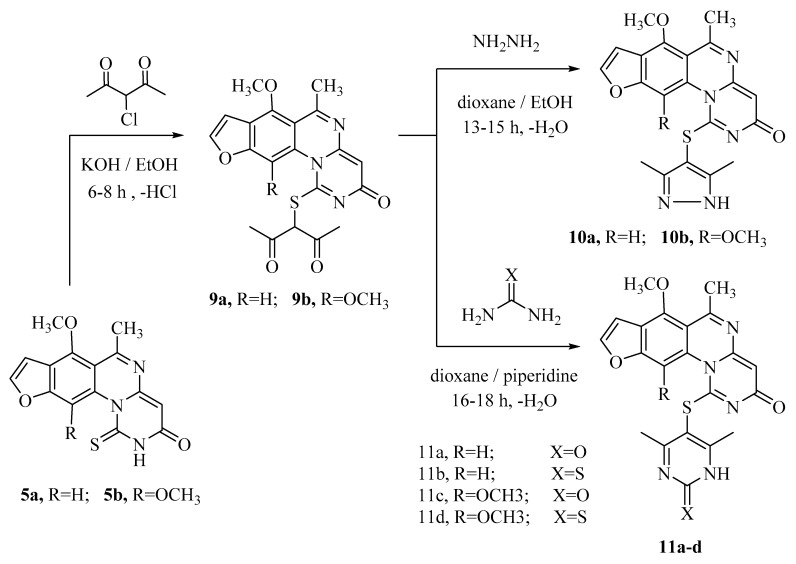
Synthesis of pyrazole, pyrimidine, -furopyrimido quinazolinone derivatives.

Alkylation of an ethanolic potassium hydroxide solution of **5a** or **5b** with 3-chloro-pentane-2,4-dione(3-chloroacetylacetone) [30], yielded the 3-(((7-methoxy or 7,11-dimethoxy)-6-methyl-3-oxo-3*H*-furo[3,2-g]pyrimido[1,6-a]quinazolin-1-yl)thio)pentane-2,4-dione (**9a**,**b**) in high yield. The IR spectra of **9a** and **9b** showed a strong absorption bands at **9a** = 1725, 1721, 1682 cm^−1^ and **9b** = 1728, 1722, 1684 cm^−1^ characteristic to three carbonyl groups, respectively. The ^13^C-NMR (DMSO-*d*_6_, ppm) of **9a** showed signals at 68.2, 91.3 and 96.5 for three carbon atoms of (CH), (CH, phenyl) and (CH, pyrimidine) and signals at 168.6, 184.5 and 188.2 three carbon atoms of the three carbonyl groups, and the molecular ion peaks of **9a** and **9b** at *m/z* 411 (95%) and 441 (90%), respectively. On the other hand, compounds **9a** and **9b**, as a typical 1,3-diketone condensation with each of hydrazine hydrate, urea and thiourea to afford the corresponding 1-((3,5-dimethyl-1*H*-pyrazol-4-yl)thio)-(7-methoxy or 7,11-dimethoxy)-6-methyl-3*H*-furo[3,2-g]pyrimido[1,6-a]quinazolin-3-one (**10a**,**b**) and 1-((4,6-dimethyl-2-(oxo or thioxo)-1,2-dihydropyrimidin-5-yl)thio)-(7-methoxy or 7,11-dimethoxy)-6-methyl-3*H*-furo[3,2-g]pyrimido[1,6-a]quinazolin-3-one (**11a**–**d**), respectively. IR spectrum of **10a** and **10b** showed absorption of a broad band at 3380–3360 cm^−1^ corresponding to NH group. Also, the compounds **11a**–**d** exhibited absorption bands at 3390–3370 cm^−1^ for NH group were detected in the IR spectrum. ^1^H-NMR spectrum of **10a** showed one singlet at 11.50 ppm corresponding to the one proton of NH group and compound **11a** revealed one singlet at 10.60 ppm for one proton of NH group (D_2_O exchangeable). All new compounds were proven by elemental and spectral analysis (IR, ^1^H, ^13^C-NMR and MS) which is mentioned in the experimental part (Scheme 3).

**Scheme 4:**
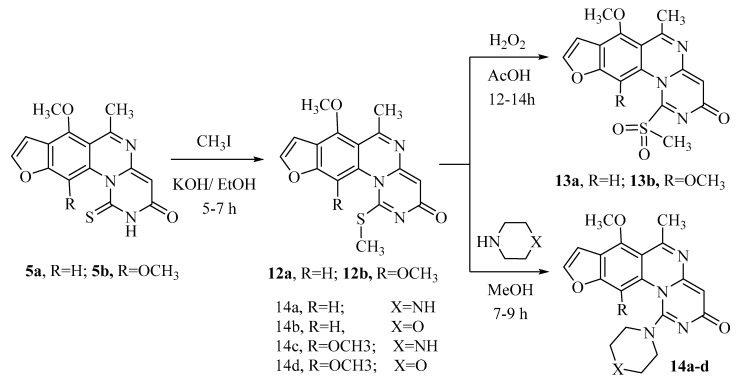
Synthesis of piperazine, morpholine, -furopyrimido-quinazolinone derivatives.

Alkylation of an ethanolic potassium hydroxide solution of **5a** or **5b** with methyl-iodide yielded the (7-methoxy or 7,11-dimethoxy)-6-methyl-1-(methylthio)-3*H*-furo[3,2-g]pyrimido[1,6-a]quinazolin-3-one (**12a**,**b**). Assignment of structures **12a** and **12b** to the reaction products is based on correct elemental analysis and IR, NMR spectroscopy are in agreement with the structure. Thus, ^1^H-NMR spectrum of **12a** or **12b** showed one singlet signal at 2.80 or 2.88 ppm indicative of three protons to (SCH_3_) group and the molecular ion peaks of **12a** or **12b** displayed at *m/z* 327 (M^+^, 100%) and 357 (M^+^, 100%), respectively. Moreover, oxidation of **12a** or **12b** with hydrogen peroxide in acetic acid yielded the (7-methoxy or 7,11-dimethoxy)-6-methyl-1-(methylsulfonyl)-3*H*-furo[3,2-g]pyrimido[1,6-a]quinazolin-3-one (**13a**,**b**). The IR spectrum of **13a** exposed the presence of two bands at 1162, 1340 cm^−1^ corresponding to (SO_2_) group, and ^1^H-NMR spectrum of **13a** exhibited one singlet at 2.95 agreeing to the three protons of (SO_2_CH_3_) group. Furthermore, heating under refluxing compounds **12a** or **12b** with secondary aliphatic amines [21,22,30], namely piperazine or morpholine in methanol, produced the (7-methoxy or 7,11-dimethoxy)-6-methyl-1-((piperazin-1-yl) or morpholino)-3*H*-furo[3,2-g]pyrimido[1,6-a]quinazolin-3-one (**14a**–**d**). IR spectrum of **14a** displayed absorption bands at 3390, 1687 cm^−1^ conforming to NH and carbonyl group, respectively. Additionally, the ^1^H-NMR spectrum of **14c** displayed a singlet broad signal at 10.35 ppm conforming to the one proton of the one (NH) group, which was D_2_O exchangeable. The mass spectrum of **13a**, **13b**, **14a**, **14b**, **14c** and **14d** revealed molecular ion peaks at *m/z* 359 (M^+^, 84%), 389 (M^+^, 80%), 365 (M^+^, 83%), 366 (M^+^, 78%), 395 (M^+^, 76%) and 396 (M^+^, 74%), respectively (Scheme 4).

### 2.2. Biological Screening

The antimicrobial activity of the compounds was tested in vitro and the results are shown in Table 1 and Table 2. Some of these compounds indicated highest antimicrobial activity, comparable to cefotaxime sodium (MIC = 2–5 μmol mL^−1^). Compounds **8a**–**f**, **11a**–**d** and **10a**–**b** displayed potent antimicrobial activity against Gram-positive bacteria; *Staphylococcus aureus* (ATCC^®^6538™), *Streptococcus pyogenes* (ATCC^®^19615™) and Gram-negative bacteria; *Escherichia coli* (ATCC^®^25922™), *Klebsiella pneumoniae* (ATCC^®^ 10031™). Also, another compounds; **14a**–**d**, **7a**–**b** and **9a**–**b** exhibited moderate antimicrobial activities. The MIC values in μmol mL^−1^ of these compounds were as the following, **8a**–**f** (1–8), **11 a**–**d** (6–10), **10a**–**b** (9–12). Compounds **8a**–**f** and **11a**–**d** exposed too higher antifungal activity with MIC in μmol/cm^3^ of **8a**–**f** (2–9) and **11a**–**d** (7–12) whose results were comparable with the positive control, nystatin (MIC 2-4 μmol mL^−1^). Some of compounds revealed moderate anti-fungal activity when compared with the nystatin (MIC 2–4 μmol mL^−1^): **10a**–**b** (10–14), **14a**–**d** (12–18), **7a**–**b** (15–20) and **9a**–**b** (17–22). The tested fungi were *Aspergillus niger* (ATCC^®^ 16888™), *Alternaria alternate*, *Curvularia lunata* and *Candida albicans* (ATCC^®^10231™). Thru comparing the observed antimicrobial activities of the furopyrimido quinazolinones, furothiazolo pyrimido quinazolinones obtained in this study to their structures, the (SAR’s) were suggested; the presence of the functional groups linked with visnagenone **2a** or khellinone **2b** derivatives, such as thioxo, methyl, hydroxyl, methoxy, amino, chloro, substituted-benzylidene, thiazole, quinazoline, pyrimidine, pyrazole, methylsulfonyl, piperazine and morpholine moieties. Remainder the compounds demonstrated a weak activity compared to the antimicrobial activity of the standard drugs (cefotaxime sodium and nystatin). The thiazolopyrimidine derivatives possessing verified to be effective as antimicrobial agents in previous articles [7,8]. Also, they were active against *Staphylococcus aureus, E. coli,* and *Klebsiella pneumoniae* [7,31] and they were also effective against *Streptococcus pyogenes* and species of fungi, namely, *Aspergillus niger, Candida albicans* [7,32]. In this work and previous reports, most bacteria and fungi types effected susceptible to this class of heterocyclic compounds [33,34,35]. Moreover, previous results and our findings corroboration the promising antimicrobial activity of furothiazolopyrimidoquinazolinone derivatives which can be developed to higher antimicrobial activities

## 3. Experimental Section

### 3.1. General Information

All melting points were taken on an Electrothermal IA 9100 series digital melting point apparatus (Shimadzu, Tokyo, Japan). Elemental analyses were performed on Vario EL (Elementar, Langenselbold, Germany). Microanalytical data were processed in the microanalytical center, Faculty of Science, Cairo University and National Research Centre. The IR spectra (KBr disc) were recorded using a Perkin-Elmer 1650 spectrometer (Waltham, MA, USA). NMR spectra were determined using JEOL 270 MHz and JEOL JMS-AX 500 MHz (JEOL, Tokyo, Japan) spectrometers with Me_4_Si as an internal standard. Mass spectra were recorded on an EI Ms-QP 1000 EX instrument (Shimadzu, Japan) at 70 eV. Biological evaluations were done by the antimicrobial unit, Department of Chemistry of Natural and Microbial Products, National Research Centre, Egypt. All starting materials and solvents were purchased from Sigma-Aldrich (Saint Louis, MO, USA).

### 3.2. General Procedure for Synthesis of 6-((1-(6-Hydroxy-(4-methoxy or 4,7-dimethoxy)-benzofuran-5-yl)ethylidene)amino)-2-thioxo-2,3-dihydropyrimidin-4(1H)-one (***4a***,***b***)

A mixture of visnaginone **2a** (2.06 g, 0.01 mol) or khellinone **2b** (2.36 g, 0.01 mol) with 6-amino-2-thioxo-2,3-dihydropyrimidin-4(1*H*)-one **3** (1.43 g, 0.01 mol) in dimethylformamide (40 mL) was refluxed for 4–6 h. The solid formed was filtered off, dried, and crystallized from the proper solvent to give **4a** and **4b**, respectively.

### 3.3. Synthesis of 6-((1-(6-Hydroxy-4-methoxybenzofuran-5-yl)ethylidene)amino)-2-thioxo-2,3-dihydro pyrimidin-4(1H)-one (***4a***)

The compound was obtained from the reaction of visnaginone (**2a**) with 6-amino-thiouracil (**3**) as yellow crystals, crystallized from methanol in 90% yield, m.p. 231 °C. IR (ν, cm^−1^) KBr: 3370 (brs, 2NH), 3030 (CH-aryl), 2960 (CH-aliph), 1685 (CO, amide), 1630 (C=N). ^1^H-NMR (DMSO-*d*_6_, ppm) δ 2.38 (s, 3H, CH_3_), 3.92 (s, 3H, OCH_3_), 6.79 (d, 1H, *J* = 2.35 Hz, furan), 7.80 (d, 1H, *J* = 2.38 Hz, furan), 7.92 (s, 1H, phenyl), 7.94 (s, 1H, pyrimidine), 10.80, 11.19 (br, 2H, 2NH, D_2_O exchangeable), 16.11 (br, 1H, OH, D_2_O exchangeable); ^13^C-NMR (DMSO-*d*_6_) δ 21.3 (1C, CH_3_), 61.7 (1C, OCH_3_), 93.1 (1C, CH, pyrimidine), 98.9 (1C, CH, phenyl), 100.5, 104.5, 108.9, 146.6, 146.9, 154.5, 160.9, 163.2, (8C, Ar-C), 164.1 (1C, C=N-ph), 168.1(1C, C=O), 173.5 (1C, C=S); MS (70 eV, %) *m/z* 331 (M^+^, 100%); Anal. Calc. (Found) for C_15_H_13_N_3_O_4_S (331.35): C, 54.37 (54.30); H, 3.95 (3.91); N, 12.68(12.60).

### 3.4. Synthesis of 6-((1-(6-Hydroxy-4,7-dimethoxybenzofuran-5-yl)ethylidene)amino)-2-thioxo-2,3-dihydro pyrimidin-4(1H)-one (***4b***)

The compound was obtained from the reaction of khellinone (**2b**) with 6-amino-thiouracil (**3**) as yellowish crystals, crystallized from dioxane in 84% yield, m.p. 211 °C. IR (ν, cm^−1^) KBr: 3371(brs, 2NH), 3032 (CH-aryl), 2965 (CH-aliph), 1682 (CO, amide), 1631 (C=N). ^1^H-NMR (DMSO-*d*_6_, ppm) δ 2.39 (s, 3H, CH_3_), 3.94 (s, 6H, 2OCH_3_), 6.80 (d, 1H, *J* = 2.34 Hz, furan), 7.83 (d, 1H, *J* = 2.37 Hz, furan), 7.95 (s, 1H, pyrimidine), 10.81,11.20 (br, 2H, 2NH, D_2_O exchangeable), 16.12 (br, 1H, OH, D_2_O exchangeable); ^13^C-NMR (DMSO-*d*_6_) δ 21.4 (1C, CH_3_), 61.6 (2C, OCH_3_), 93.2 (1C, CH, pyrimidine), 100.5, 104.7, 109.9, 129.3, 146.2, 147.1, 153.8, 158.9, 164.7, (9C, Ar-C), 165.2 (1C, C=N-ph), 167.9 (1C, C=O), 173.8 (1C, C=S); MS (70 eV, %) *m/z* 361 (M^+^, 100%); Anal. Calc. (Found) for C_16_H_15_N_3_O_5_S (361.37): C, 53.18 (53.23); H, 4.18 (4.13); N, 11.63 (11.68).

### 3.5. General Procedure for Synthesis of (7-Methoxy or 7,11-dimethoxy)-6-methyl-1-thioxo-1,2-dihydro-3H-furo[3,2-g]pyrimido[1,6-a]quinazolin-3-one (***5a***,***b***)

Method A: A mix of visnaginone **2a** (2.06 g, 0.01 mol) or khellinone **2b** (2.36 g, 0.01 mol) with 6-amino-2-thiouracil (**3**) in DMF (40 mL) was refluxed for 14–16 h. The product precipitated was filtered off and washed with 100 mL water, dried and crystallized from the proper solvent to give **5a** and **5b**, respectively. Method B: A refluxing of **4a** (3.31 g, 0.01 mol) or **4b** (3.61 g, 0.01 mol) in DMF (40 mL) was refluxed for 8–10 h under control (TLC). The final precipitated was filtered off and washed with 50 mL ethanol, dried, and crystallized from the proper solvent to give **5a** and **5b**, respectively.

### 3.6. Synthesis of 7-Methoxy-6-methyl-1-thioxo-1,2-dihydro-3H-furo[3,2-g]pyrimido[1,6-a]quinazolin-3-one (***5a***)

The compound was obtained from the reaction of visnaginone (**2a**) with 6-aminothiouracil (**3**) or compound (**4a**) in dimethylformamide, as yellowish crystals, crystallized from benzene in 85% yield, m.p. 271 °C. IR (ν, cm^−1^) KBr: 3350 (brs, NH), 3028 (CH-aryl), 2955 (CH-aliph), 1680 (CO, amide), 1635 (C=N). ^1^H-NMR (DMSO-*d*_6_, ppm) δ 2.32 (s, 3H, CH_3_), 3.92 (s, 3H, OCH_3_), 6.75 (d, 1H, *J* = 2.33 Hz, furan), 7.33 (s, 1H, pyrimidine), 7.80 (d, 1H, *J* = 2.36 Hz, furan), 8.29 (s, 1H, phenyl), 11.20 (br, 1H, NH, D_2_O exchangeable); ^13^C-NMR (DMSO-*d*_6_) δ 24.5 (1C, CH_3_), 61.8 (1C, OCH_3_), 79.7 (1C, CH, pyrimidine), 99.1 (1C, CH, phenyl), 104.9, 108.9,116.6, 140.9, 146.6, 148.9, 160.1, 161.2, 164.1 (9C, Ar-C), 168.2 (1C, C=O), 173.6 (1C, C=S); MS (70 eV, %) *m/z* 313 (M^+^, 100%); Anal. Calc. (Found) for C_15_H_11_N_3_O_3_S (313.33): C, 57.50 (57.58); H, 3.54 (3.59); N, 13.41(13.50).

### 3.7. Synthesis of 7,11-Dimethoxy-6-methyl-1-thioxo-1,2-dihydro-3H-furo[3,2-g]pyrimido[1,6-a]quinazolin-3-one (***5b***)

The compound was obtained from the reaction of khellinone (**2b**) with 6-amino- thiouracil (**3**) or compound (**4b**) in dimethylformamide, as yellow crystals, crystallized from toluene in 82% yield, m.p. 251 °C. IR (ν, cm^−1^) KBr: 3352 (brs, NH), 3029 (CH-aryl), 2957 (CH-aliph), 1681 (CO, amide), 1633 (C=N). ^1^H-NMR (DMSO-*d*_6_, ppm) δ 2.35 (s, 3H, CH_3_), 3.95 (s, 6H, 2OCH_3_), 6.77 (d, 1H, *J* = 2.31 Hz, furan), 7.30 (s, 1H, pyrimidine), 7.82 (d, 1H, *J* = 2.37 Hz, furan), 11.25 (br, 1H, NH, D_2_O exchangeable); ^13^C-NMR (DMSO-*d*_6_) δ 24.6 (1C, CH_3_), 61.9 (2C, 2OCH_3_), 80.2 (1C, CH, pyrimidine), 105.1, 109.2, 115.9, 125.8, 137.5, 142.8, 146.5, 149.2, 161.7, 164.8 (10C, Ar-C), 168.4 (1C, C=O), 173.8 (1C, C=S); MS (70 eV, %) *m/z* 343 (M^+^, 100%); Anal. Calc. (Found) for C_16_H_13_N_3_O_4_S (343.36): C, 55.97 (55.90); H, 3.82 (3.75); N, 12.24 (12.29).

### 3.8. General Procedure for Synthesis of 2-(((7-Methoxy or 7,11-dimethoxy)-6-methyl-3-oxo-3H-furo[3,2-g]pyrimido[1,6-a]quinazolin-1-yl)thio) Acetic Acid (***6a***,***b***)

A mixture from **5a** (3.13 g, 0.01 mol) or **5b** (3.43 g, 0.01 mol), chloroacetic acid (0.94 g, 0.01 mol) and (0.02 mol) of anhydrous sodium acetate was stirred under reflux in 40 mL of glacial acetic acid and 20 mL of acetic anhydride for 3–5 h. The reaction mixture was cooled and poured into cold water (100 mL). The deposited precipitate was filtered off, and crystallized from appropriate solvent to produce **6a** and **6b**, respectively.

### 3.9. Synthesis of 2-((7-Methoxy-6-methyl-3-oxo-3H-furo[3,2-g]pyrimido[1,6-a]quinazolin-1-yl)thio) Acetic Acid (***6a***)

The compound was obtained from the reaction of (**5a**) with chloroacetic acid, as brownish crystals, crystallized from hexane in 88% yield, m.p. 294 °C. IR (ν, cm^−1^) KBr: 3340 (brs, OH), 3025 (CH-aryl), 2950 (CH-aliph), 1745 (CO, acid), 1684 (CO, amide), 1631 (C=N). ^1^H-NMR (DMSO-*d*_6_, ppm) δ 2.30 (s, 3H, CH_3_), 3.91 (s, 3H, OCH_3_), 4.27 (s, 2H, CH_2_), 6.77 (d, 1H, *J* = 2.32 Hz, furan), 7.35 (s, 1H, pyrimidine), 7.78 (d, 1H, *J* = 2.35 Hz, furan), 7.90 (s, 1H, phenyl), 13.70 (br, 1H, OH, D_2_O exchangeable); ^13^C-NMR (DMSO-*d*_6_) δ 23.6 (1C, CH_3_), 32.8 (1C, CH_2_), 61.7 (1C, OCH_3_), 97.2 (1C, CH, phenyl), 100.6 (1C, CH, pyrimidine), 103.4, 105.3, 108.5, 143.7, 146.5, 152.2,158.4, 160.6, 163.9,164.8 (10C, Ar-C), 169.1, 172.8 (2C, 2C=O); MS (70 eV, %) *m/z* 371 (M^+^, 100%); Anal. Calc. (Found) for C_17_H_13_N_3_O_5_S (371.37): C, 54.98 (54.91); H, 3.53 (3.58); N, 11.32(11.40).

### 3.10. Synthesis of 2-((7,11-Dimethoxy-6-methyl-3-oxo-3H-furo[3,2-g]pyrimido[1,6-a]quinazolin-1-yl)thio) Acetic Acid (***6b***)

The compound was obtained from the reaction of (**5b**) with chloroacetic acid, as yellowish crystals, crystallized from ethanol in 86% yield, m.p. 283 °C. IR (ν, cm^−1^) KBr: 3345 (brs, OH), 3027 (CH-aryl), 2952 (CH-aliph), 1748 (CO, acid), 1681 (CO, amide), 1632 (C=N). ^1^H-NMR (DMSO-*d*_6_, ppm) δ 2.31 (s, 3H, CH_3_), 3.95 (s, 6H, 2OCH_3_), 4.25 (s, 2H, CH_2_), 6.79 (d, 1H, *J* = 2.35 Hz, furan), 7.38 (s, 1H, pyrimidine), 7.80 (d, 1H, *J* = 2.35 Hz, furan), 13.75 (br, 1H, OH, D_2_O exchangeable); ^13^C-NMR (DMSO-*d*_6_) *δ* 23.4 (1C, CH_3_), 32.6 (1C, CH_2_), 61.3 (2C, 2OCH_3_), 100.1 (1C, CH, pyrimidine), 101.2, 105.4, 108.8, 124.1, 130.1, 146.2, 146.7, 150.5, 160.1, 164.4, 166.9 (11C, Ar-C), 169.5, 172.9 (2C, 2C=O); MS (70 eV, %) *m/z* 401 (M^+^, 100%); Anal. Calc. (Found) for C_18_H_15_N_3_O_6_S (401.39): C, 53.86 (53.80); H, 3.77 (3.71); N, 10.47 (10.42).

### 3.11. General Procedure for Synthesis of (9-Methoxy or 9,13-dimethoxy)-8-methyl-2-hydro-5H,14aH-furo[3,2-g]thiazolo[2′,3′:2,3] pyrimido[1,6-a]quinazoline-3,5-dione (***7a***,***b***)

Method A: A mixture from **5a** (3.13 g, 0.01 mol) or **5b** (3.43 g, 0.01 mol), chloroacetic acid (0.94 g, 0.01 mol) and (0.02 mol) of anhydrous sodium acetate was stirred under reflux in 40 mL of glacial acetic acid and 20 mL of acetic anhydride on a water bath (60–70 °C) for 13–15 h (TLC). The reaction mixture was allowed to cool to room temperature and poured into water (100 mL). The solid precipitate was filtered off, and crystallized from appropriate solvent to produced **7a** and **7b** in good yields, respectively. Method B: A mixture of **6a** (3.71 g, 0.01 mol) or **6b** (4.01 g, 0.01 mol) in dimethylformamide (35 mL) was refluxed for 6–8 h with (TLC). The final product was filtered off, dried and crystallized from the proper solvent to give **7a** and **7b**, respectively.

### 3.12. Synthesis of 9-Methoxy-8-methyl-2-hydro-5H,14aH-furo[3,2-g]thiazolo[2′,3′:2,3]pyrimido[1,6-a]quinazoline-3,5-dione (***7a***)

The compound was obtained from the reaction of (**5a**) with chloroacetic acid or compound (**6a**) in DMF, as white crystals, crystallized from dioxane in 82% yield, m.p. 338 °C. IR (ν, cm^−1^) KBr: 3035 (CH-aryl), 2962 (CH-aliph), 1688, 1680 (2CO, amide), 1635 (C=N). ^1^H-NMR (DMSO-*d*_6_, ppm) δ 2.37 (s, 3H, CH_3_), 3.93 (s, 3H, OCH_3_), 4.21 (s, 2H, CH_2_), 5.57 (s, 1H, CH, thiazole), 6.78 (d, 1H, *J* = 2.34 Hz, furan), 7.38 (s, 1H, pyrimidine), 7.75 (d, 1H, *J* = 2.32 Hz, furan), 7.88 (s, 1H, phenyl); ^13^C-NMR (DMSO-*d*_6_) *δ* 23.3 (1C, CH_3_), 32.6 (1C, CH_2_), 61.8 (1C, OCH_3_), 76.1 (1C, CH, thiazole), 94.5 (1C, CH, phenyl), 98.8 (1C, CH, pyrimidine), 101.2, 105.5, 107.9, 142.2, 146.4, 153.5, 159.7, 160.4, 164.5 (9C, Ar-C), 166.4, 170.1 (2C, 2C=O); MS (70 eV, %) *m/z* 355 (M^+^, 90%); Anal. Calc. (Found) for C_17_H_13_N_3_O_4_S (355.37): C, 57.46 (57.40); H, 3.69 (3.62); N, 11.82 (11.88).

### 3.13. Synthesis of 9,13-Dimethoxy-8-methyl-2-hydro-5H,14aH-furo[3,2-g]thiazolo[2′,3′:2,3]pyrimido[1,6-a]quinazoline-3,5-dione (***7b***)

The compound was obtained from the reaction of (**5b**) with chloroacetic acid or compound (**6b**) in DMF, as yellowish crystals, crystallized from methanol in 80% yield, m.p. 303 °C. IR (ν, cm^−1^) KBr: 3038 (CH-aryl), 2966 (CH-aliph), 1685, 1682 (2CO, amide), 1632 (C=N). ^1^H-NMR (DMSO-*d_6_*, ppm) δ 2.33 (s, 3H, CH_3_), 3.97 (s, 6H, 2OCH_3_), 4.24 (s, 2H, CH_2_), 5.62 (s, 1H, CH, thiazole), 6.74 (d, 1H, *J* = 2.33 Hz, furan), 7.39 (s, 1H, pyrimidine), 7.77 (d, 1H, *J* = 2.31 Hz, furan); ^13^C-NMR (DMSO-*d_6_*) δ 23.1 (1C, CH_3_), 32.3 (1C, CH_2_), 61.7 (2C, 2OCH_3_), 76.4 (1C, CH, thiazole), 97.5 (1C, CH, pyrimidine), 101.4, 105.6, 108.2, 123.6, 127.8, 146.3, 146.8, 152.9, 160.1, 164.7 (10C, Ar-C), 166.2, 170.5 (2C, 2C=O); MS (70 eV, %) *m*/*z* 385 (M^+^, 95%); Anal. Calc. (Found) for C_18_H_15_N_3_O_5_S (385.39): C, 56.10 (56.18); H, 3.92 (3.85); N, 10.90 (10.98).

### 3.14. General Procedure for Synthesis of 2-(Substituted-benzylidene)-9,(substituted)-methoxy-8-methyl-2-hydro-5H,14aH-furo[3,2-g]thiazolo[2′,3′:2,3]pyrimido[1,6-a]quinazoline-3,5-dione (***8a***–***f***)

Method A: One pot synthesis: A mixture from **5a** (3.13g, 0.01 mol) or **5b** (3.43 g, 0.01 mol), chloroacetic acid (0.94 g, 0.01 mol), the appropriate aromatic aldehyde (10 mmol) and (0.02 mol) of anhydrous sodium acetate was stirred under reflux in 40 mL of glacial acetic acid and 20 mL of acetic anhydride for 24–26 h. The reaction mixture was cooled and poured into ice water. The deposited precipitate was filtered off, and crystallized from appropriate solvent to give (**8a**–**f**).

Method B: A mixture of compound **7a** (3.55 g, 10 mmol) or **7b** (3.85 g, 10 mmol) and the appropriate aromatic aldehyde (10 mmol) in dioxane (40 mL) containing a catalyst amount of piperidine (0.5 mL) was stirred and heated under reflux for 10–12 h (TLC control). The reaction mixture was cooled, the formed precipitate filtered off, dried and recrystallized from the appropriate solvent to afford (**8a**–**f**).

### 3.15. Synthesis of 2-Benzylidene-9-methoxy-8-methyl-2-hydro-5H,14aH-furo[3,2-g]thiazolo[2′,3′:2,3] pyrimido[1,6-a]quinazoline-3,5-dione (***8a***)

The compound was obtained from the reaction of (**5a**) and benzaldehyde (1.06 g, 10 mmol) with chloroacetic acid or compound (**7a**) with benzaldehyde, as yellowish crystals, crystallized from dimethylformamide in 75% yield, m.p. > 350 °C. IR (ν, cm^−1^) KBr: 3050 (CH-aryl), 2945 (CH-aliph), 1685, 1672 (2CO, amide), 1630 (C=N). ^1^H-NMR (DMSO-*d*_6_, ppm) δ 2.30 (s, 3H, CH_3_), 3.91 (s, 3H, OCH_3_), 5.59 (s, 1H, CH, thiazole), 6.75 (d, 1H, *J* = 2.31 Hz, furan), 7.32 (s, 1H, pyrimidine), 7.40–7.67(m, 5H, phenyl), 7.72 (d, 1H, *J* = 2.30 Hz, furan), 7.85 (s, 1H, phenyl), 8.05 (s, 1H, CH); ^13^C-NMR (DMSO-*d*_6_) δ 23.1 (1C, CH_3_), 61.6 (1C, OCH_3_), 77.5 (1C, CH, thiazole), 90.8 (1C, CH, phenyl), 92.9 (1C, CH, pyrimidine), 100.7, 104.8, 105.6, (3C, benzofurane), 121.9 (1C, CH), 127.2, 128.1, 128.7, 133.9 (6C, phenyl), 137.5 (1C, thiazole), 140.3, 146.4, 153.8, 160.1, 160.4, 164.2 (6C, pyrimidobenzo furane), 165.6, 168.4 (2C, 2C=O); MS (70 eV, %) *m/z* 443 (M^+^, 88%); Anal. Calc. (Found) for C_24_H_17_N_3_O_4_S (443.48): C, 65.00 (65.10); H, 3.86 (3.80); N, 9.48 (9.41).

### 3.16. Synthesis of 2-(4-Chlorobenzylidene)-9-methoxy-8-methyl-2-hydro-5H,14aH-furo[3,2-g]thiazolo[2′,3′:2,3]pyrimido[1,6-a]quinazoline-3,5-dione (***8b***)

The compound was obtained from the reaction of (**5a**) and 4-chlorobenzaldehyde (1.40 g, 10 mmol) with chloroacetic acid or compound (**7a**) with 4-chloro- benzaldehyde, as yellow crystals, crystallized from dioxane in 80% yield, m.p. > 350 °C. IR (ν, cm^−1^) KBr: 3052 (CH-aryl), 2948 (CH-aliph), 1687, 1674 (2CO, amide), 1631 (C=N). ^1^H-NMR (DMSO-*d*_6_, ppm) δ 2.31 (s, 3H, CH_3_), 3.90 (s, 3H, OCH_3_), 5.60 (s, 1H, CH, thiazole), 6.74 (d, 1H, *J* = 2.32 Hz, furan), 7.33 (s, 1H, pyrimidine), 7.45–7.50 (dd, 2H, *J* = 7.60, 7.64 Hz, 4-chlorophenyl), 7.55–7.60 (dd, 2H, *J* = 7.62, 7.66 Hz, 4-chloro phenyl),7.70 (d, 1H, *J* = 2.34 Hz, furan), 7.83 (s, 1H, phenyl), 8.08 (s, 1H, CH); ^13^C-NMR (DMSO-*d*_6_) δ 23.2 (1C, CH_3_), 61.4 (1C, OCH_3_), 77.8 (1C, CH, thiazole), 90.2 (1C, CH, phenyl), 92.1 (1C, CH, pyrimidine), 100.3, 104.9, 105.5, (3C, benzofurane), 122.1 (1C, CH), 128.4, 129.2, 132.5, 132.8 (6C, 4-chlorophenyl), 137.9 (1C, thiazole), 140.6, 146.3,153.5, 160.3, 160.7, 164.3 (6C, pyrimidobenzo furane), 165.8, 168.7 (2C, 2C=O); MS (70 eV, %) *m/z* 477 (M^+^, 90%); Anal. Calc. (Found) for C_24_H_16_ClN_3_O_4_S (477.92): C, 60.32 (60.39); H, 3.37 (3.44); N, 8.79 (8.85).

### 3.17. Synthesis of 9-Methoxy-2-(4-methoxybenzylidene)-8-methyl-2-hydro-5H,14aH-furo[3,2-g]thiazolo[2′,3′:2,3]pyrimido[1,6-a]quinazoline-3,5-dione (***8c***)

The compound was obtained from the reaction of (**5a**) and 4-methoxy- benzaldehyde (1.36 g, 10 mmol) with chloroacetic acid or compound (**7a**) with 4-methoxybenzaldehyde, as brownish crystals, crystallized from ethanol in 78% yield, m.p. > 350 °C. IR (ν, cm^−1^) KBr: 3054 (CH-aryl), 2944 (CH-aliph), 1683, 1671 (2CO, amide), 1636 (C=N). ^1^H-NMR (DMSO-*d*_6_, ppm) δ 2.29 (s, 3H, CH_3_), 3.92 (s, 3H, OCH_3_), 4.10 (s, 3H, OCH_3_), 5.59 (s, 1H, CH, thiazole), 6.71 (d, 1H, *J* = 2.32 Hz, furan), 7.28 (s, 1H, pyrimidine), 7.48–7.53 (dd, 2H, *J* = 7.61, 7.65 Hz, 4-methoxyphenyl), 7.58–7.63 (dd, 2H, *J* = 7.63, 7.67 Hz, 4-methoxyphenyl), 7.69 (d, 1 H, *J* = 2.37 Hz, furan), 7.86 (s, 1 H, phenyl), 8.06 (s, 1H, CH); ^13^C-NMR (DMSO-*d*_6_) δ 23.4 (1C, CH_3_), 58.5 (1C, OCH_3_), 61.7 (1C, OCH_3_), 77.2 (1C, CH, thiazole), 90.5 (1C, CH, phenyl), 92.4 (1C, CH, pyrimidine), 100.4, 105.1, 105.5, (3C, benzofurane), 122.6 (1C, CH), 123.1, 127.7, 130.1, 155.5 (6C, 4-methoxyphenyl), 137.4 (1C, thiazole), 140.8, 146.2,153.6, 160.1, 160.4, 164.3 (6C, pyrimidobenzofurane), 165.1, 168.6 (2C, 2C=O); MS (70 eV, %) *m/z* 473 (M^+^, 87%); Anal. Calc. (Found) for C_25_H_19_N_3_O_5_S (473.50): C, 63.42 (63.49); H, 4.04 (4.12); N, 8.87 (8.95).

### 3.18. Synthesis of 2-Benzylidene-9,13-dimethoxy-8-methyl-2-hydro-5H,14aH-furo[3,2-g]thiazolo [2′,3′:2,3]pyrimido[1,6-a]quinazoline-3,5-dione (***8d***)

The compound was obtained from the reaction of (**5b**) and benzaldehyde (1.06 g, 10 mmol) with chloroacetic acid or compound (**7b**) with benzaldehyde, as yellow crystals, crystallized from acetone in 79% yield, m.p. > 350 °C. IR (ν, cm^−1^) KBr: 3060 (CH-aryl), 2950 (CH-aliph), 1688, 1678 (2CO, amide), 1638 (C=N). ^1^H-NMR (DMSO-*d*_6_, ppm) δ 2.33 (s, 3H, CH_3_), 3.97 (s, 6H, 2OCH_3_), 5.60 (s, 1H, CH, thiazole), 6.74 (d, 1H, *J*= 2.30 Hz, furan), 7.34 (s, 1H, pyrimidine), 7.45–7.65 (m, 5H, phenyl), 7.74 (d, 1H, *J* = 2.35 Hz, furan), 8.02 (s, 1H, CH); ^13^C-NMR (DMSO-*d*_6_) δ 23.5 (1C, CH_3_), 61.8 (2C, 2OCH_3_), 77.7 (1C, CH, thiazole), 91.7 (1C, CH, pyrimidine), 101.4, 105.3, 108.8, 120.7 (4C, benzo furane), 122.2 (1C, CH), 126.1 (1C, pyrimidine), 127.7, 128.3, 128.8, 134.6 (6C, phenyl), 137.7 (1C, thiazole), 146.2, 146.7, 151.9, 160.6, 164.8 (5C, pyrimidobenzofurane), 165.1, 168.3 (2C, 2C=O); MS (70 eV, %) *m/z* 473 (M^+^, 84%); Anal. Calc. (Found) for C_25_H_19_N_3_O_5_S (473.50): C, 63.42 (63.50); H, 4.04 (4.10); N, 8.87 (8.80).

### 3.19. Synthesis of 2-(4-Chlorobenzylidene)-9,13-dimethoxy-8-methyl-2-hydro-5H,14aH-furo[3,2-g]thiazolo [2′,3′:2,3]pyrimido[1,6-a]quinazoline-3,5-dione (***8e***)

The compound was obtained from the reaction of (**5b**) and 4-chlorobenzaldehyde (1.40 g, 10 mmol) with chloroacetic acid or compound (**7b**) with 4-chlorobenzaldehyde, as yellowish crystals, crystallized from methanol in 88% yield, m.p. > 350 °C. IR (ν, cm^−1^) KBr: 3055 (CH-aryl), 2945 (CH-aliph), 1686, 1674 (2CO, amide), 1634 (C=N). ^1^H-NMR (DMSO-*d*_6_, ppm) δ 2.30 (s, 3H, CH_3_), 3.98 (s, 6H, 2OCH_3_), 5.59 (s, 1H, CH, thiazole), 6.73 (d, 1H, *J*= 2.31 Hz, furan), 7.35 (s, 1H, pyrimidine), 7.47–7.52 (dd, 2H, *J* = 7.68, 7.62 Hz, 4-chlorophenyl), 7.57–7.62 (dd, 2H, *J* = 7.61, 7.65 Hz, 4-chloro phenyl), 7.75 (d, 1H, *J* = 2.36 Hz, furan), 8.04 (s, 1H, CH); ^13^C-NMR (DMSO-*d*_6_) δ 23.7 (1C, CH_3_), 61.9 (2C, 2OCH_3_), 77.6 (1C, CH, thiazole), 91.2 (1C, CH, pyrimidine), 100.5, 105.6, 108.4, 120.8 (4C, benzo furane), 122.4 (1C, CH), 126.3 (1C, pyrimidine), 128.2, 128.8, 131.5, 131.9 (6C, 4-chloro phenyl), 137.5 (1C, thiazole), 146.3, 146.9, 150.8, 160.4, 164.9 (5C, pyrimidobenzofurane), 165.3, 168.1 (2C, 2C=O); MS (70 eV, %) *m/z* 507 (M^+^, 98%); Anal. Calc. (Found) for C_25_H_18_ClN_3_O_5_S (507.94): C, 59.12 (59.22); H, 3.57 (3.50); N, 8.27 (8.35).

### 3.20. Synthesis of 9,13-Dimethoxy-2-(4-methoxybenzylidene)-8-methyl-2-hydro-5H,14aH-furo[3,2-g]thiazolo [2′,3′:2,3]pyrimido[1,6-a]quinazoline-3,5-dione (***8f***)

The compound was obtained from the reaction of (**5b**) and 4-methoxy- benzaldehyde (1.36 g, 10 mmol) with chloroacetic acid or compound (**7b**) with 4-methoxybenzaldehyde, as brownish crystals, crystallized from benzene in 73% yield, m.p. > 350 °C. IR (ν, cm^−1^) KBr: 3052 (CH-aryl), 2944 (CH-aliph), 1685, 1672 (2CO, amide), 1630 (C=N). ^1^H-NMR (DMSO-*d*_6_, ppm) δ 2.28 (s, 3H, CH_3_), 3.95 (s, 6H, 2OCH_3_), 4.12 (s, 3H, OCH_3_), 5.55 (s, 1H, CH, thiazole), 6.71 (d, 1H, *J*= 2.32 Hz, furan), 7.37 (s, 1H, pyrimidine), 7.49–7.54 (dd, 2H, *J* = 7.65, 7.61 Hz, 4-methoxyphenyl), 7.58–7.63 (dd, 2H, *J* = 7.62, 7.66 Hz, 4-methoxyphenyl), 7.76 (d, 1H, *J* = 2.35 Hz, furan), 8.06 (s, 1H, CH); ^13^C-NMR (DMSO-*d*_6_) δ 23.4 (1C, CH_3_), 58.2 (1C, OCH_3_), 61.8 (2C, 2OCH_3_), 77.3 (1C, CH, thiazole), 91.5 (1C, CH, pyrimidine), 100.2, 105.5, 108.6, 120.5 (4C, benzofurane), 122.7 (1C, CH), 126.1 (1C, pyrimidine), 127.2, 128.4, 129.1, 152.2 (6C, 4-methoxyphenyl), 137.8 (1C, thiazole), 146.1, 146.7, 150.6, 160.5, 164.8 (5C, pyrimidobenzo furane), 165.7, 168.2 (2C, 2C=O); MS (70 eV, %) *m/z* 503 (M^+^, 91%); Anal. Calc. (Found) for C_26_H_21_N_3_O_6_S (503.53): C, 62.02 (62.10); H, 4.20 (4.28); N, 8.35 (8.27).

### 3.21. General Procedure for Synthesis of 3-(((7-Methoxy or 7,11-dimethoxy)-6-methyl-3-oxo-3H-furo[3,2-g]pyrimido[1,6-a]quinazolin-1-yl)thio)pentane-2,4-dione (***9a***,***b***)

To a warmed ethanolic potassium hydroxide solution (prepared by dissolving 10 mmol of KOH in 50 mL ethanol) was added each of **5a** (3.13 g, 0.01 mol) or **5b** (3.43 g, 0.01 mol), the heating was continued for 40 min and the mixture was allowed to cool to room temperature, and the proper 3-chloro-pentane-2,4-dione (3-chloroacetylacetone, 1.12 mL, 0.01 mol) was added. The mixture was stirred under reflux for 6–8 h (control TLC), and then cool to room temperature, poured into cold water (100 mL). The solid product precipitated was filtered off, washed with 100 mL water; the product was dried and crystallized from the suitable solvent to afford **9a** and **9b** in good yields, respectively.

### 3.22. Synthesis of 3-((7-Methoxy-6-methyl-3-oxo-3H-furo[3,2-g]pyrimido[1,6-a]quinazolin-1-yl)thio)pentane- 2,4-dione (***9a***)

The compound was obtained from the reaction of (***5a***) with 3-chloro-pentane-2,4-dione (3-chloroacetylacetone), as white crystals, crystallized from hexane in 92% yield, m.p. > 350 °C. IR (ν, cm^−1^) KBr: 3035 (CH-aryl), 2945 (CH-aliph), 1725, 1721 (2CO, acetyl), 1682 (CO, amide), 1638 (C=N). ^1^H-NMR (DMSO-*d*_6_, ppm) *δ* 2.26 (s, 6H, 2COCH_3_), 2.34 (s, 3H, CH_3_), 3.90 (s, 3H, OCH_3_), 4.08 (s, 1H, CH), 6.73 (d, 1H, *J* = 2.31 Hz, furan), 7.37 (s, 1H, pyrimidine), 7.62 (s, 1H, phenyl), 7.75 (d, 1H, *J* = 2.33 Hz, furan), ^13^C-NMR (DMSO-*d*_6_) δ 23.8(1C, CH_3_), 26.5 (2C, 2COCH_3_), 61.6 (1C, OCH_3_), 68.2 (1C, CH), 91.3 (1C, CH, phenyl), 96.5 (1C, CH, pyrimidine), 100.1, 105.3, 108.5, 142.6, 146.1, 153.5, 157.4, 160.3, 163.8, 165.7 (10C, Ar-C), 168.6, 184.5, 188.2 (3C, 3C=O); MS (70 eV, %) *m/z* 411 (M^+^, 95%); Anal. Calc. (Found) for C_20_H_17_N_3_O_5_S (411.43): C, 58.39 (58.32); H, 4.16 (4.10); N, 10.21 (10.16).

### 3.23. Synthesis of 3-((7,11-Dimethoxy-6-methyl-3-oxo-3H-furo[3,2-g]pyrimido[1,6-a]quinazolin-1-yl)thio)pentane-2,4-dione (***9b***)

The compound was obtained from the reaction of (**5b**) with 3-chloro-pentane-2,4-dione (3-chloroacetylacetone), as white crystals, crystallized from dioxane in 90% yield, m.p. 348 °C. IR (ν, cm^−1^) KBr: 3038 (CH-aryl), 2949 (CH-aliph), 1728, 1722 (2CO, acetyl), 1684 (CO, amide), 1636 (C=N). ^1^H-NMR (DMSO-*d*_6_, ppm) δ 2.28 (s, 6H, 2COCH_3_), 2.37 (s, 3H, CH_3_), 3.98 (s, 6H, 2OCH_3_), 4.15 (s, 1H, CH), 6.72 (d, 1H, *J* = 2.34 Hz, furan), 7.35 (s, 1H, pyrimidine), 7.79 (d, 1H, *J* = 2.37 Hz, furan), ^13^C-NMR (DMSO-*d*_6_) δ 23.4 (1C, CH_3_), 26.1 (2C, 2COCH_3_), 61.9 (2C, 2OCH_3_), 68.5 (1C, CH), 97.1 (1C, CH, pyrimidine), 100.2, 105.7, 108.9, 122.5, 130.2, 146.3, 146.8, 150.7, 159.6, 164.2, 166.4 (11C, Ar-C), 168.8, 185.1, 188.7 (3C, 3C=O); MS (70 eV, %) *m/z* 441 (M^+^, 90%); Anal. Calc. (Found) for C_21_H_19_N_3_O_6_S (441.46): C, 57.14 (57.20); H, 4.34 (4.39); N, 9.52 (9.60).

### 3.24. General Procedure for Synthesis of 1-((3,5-Dimethyl-1H-pyrazol-4-yl)thio)-(7-methoxy or 7,11-dimethoxy)-6-methyl-3H-furo[3, 2-g]pyrimido[1,6-a]quinazolin-3-one (***10a***,***b***)

A mixture of **9a** (4.11 g, 0.01 mol) or **9b** (4.41 g, 0.01 mol), and hydrazine hydrate (99–100%) in dioxane (30 mL) and ethanol (20 mL) was stirred under reflux for 13–15 h. The reaction mixture was allowed to cool to room temperature, poured into cold water (100 mL). The deposited precipitate was filtered off, dried, and crystallized from the proper solvent to give **10a** and **10b** in good yields, respectively.

### 3.25. Synthesis of 1-((3,5-Dimethyl-1H-pyrazol-4-yl)thio)-7-methoxy-6-methyl-3H-furo[3,2-g]pyrimido[1,6-a]quinazolin-3-one (***10a***)

The compound was obtained from the reaction of (**9a**) with hydrazine hydrate, as yellow crystals, crystallized from dimethylformamide in 95% yield, m.p. > 350 °C. IR (ν, cm^−1^) KBr: 3375 (brs, NH), 3030 (CH-aryl), 2940 (CH-aliph), 1684 (CO, amide), 1633 (C=N). ^1^H-NMR (DMSO-*d*_6_, ppm) *δ* 2.28 (s, 3H, CH_3_), 2.38 (s, 3H, CH_3_), 2.40 (s, 3H, CH_3_), 3.92 (s, 3H, OCH_3_), 6.75 (d, 1H, *J* = 2.32 Hz, furan), 7.32 (s, 1H, pyrimidine), 7.60 (s, 1H, phenyl), 7.72 (d, 1H, *J* = 2.37 Hz, furan), 11.50 (brs, NH, D_2_O exchangeable); ^13^C-NMR (DMSO-*d*_6_) δ 20.1, 20.3, 23.2 (3C, 3CH_3_), 61.4 (1C, OCH_3_), 91.1 (1C, CH, phenyl), 96.3 (1C, CH, pyrimidine), 100.2, 105.1, 107.2, 108.4, 142.3, 144.5, 146.6, 154.1, 158.2, 160.5, 164.4, 166.3 (13C, Ar-C), 168.8 (1C, C=O); MS (70 eV, %) *m/z* 407 (M^+^, 100%); Anal. Calc. (Found) for C_20_H_17_N_5_O_3_S (407.45): C, 58.96 (58.88); H, 4.21 (4.15); N, 17.19 (17.10).

### 3.26. Synthesis of 1-((3,5-Dimethyl-1H-pyrazol-4-yl)thio)-7,11-dimethoxy-6-methyl-3H-furo[3,2-g]pyrimido[1,6-a]quinazolin-3-one (***10b***)

The compound was obtained from the reaction of (**9b**) with hydrazine hydrate, as yellowish crystals, crystallized from dioxane in 91% yield, m.p. > 350 °C. IR (ν, cm^−1^) KBr: 3370 (brs, NH), 3032 (CH-aryl), 2943 (CH-aliph), 1682 (CO, amide), 1635 (C=N). ^1^H-NMR (DMSO-*d*_6_, ppm) δ 2.30 (s, 3H, CH_3_), 2.39 (s, 3H, CH_3_), 2.41 (s, 3H, CH_3_), 3.98 (s, 6H, 2OCH_3_), 6.77 (d, 1H, *J* = 2.36 Hz, furan), 7.38 (s, 1H, pyrimidine), 7.79 (d, 1H, *J* = 2.34 Hz, furan), 11.55 (brs, NH, D_2_O exchangeable); ^13^C-NMR (DMSO-*d*_6_) δ 20.4, 20.6, 23.5 (3C, 3CH_3_), 61.9 (2C, 2OCH_3_), 96.7 (1C, CH, pyrimidine), 100.3, 105.6, 107.8, 108.5, 122.5, 130.4, 144.8, 146.1, 146.8, 151.4, 160.5, 164.2, 166.6 (14C, Ar-C), 168.9 (1C, C=O); MS (70 eV, %) *m/z* 437 (M^+^, 100%); Anal. Calc. (Found) for C_21_H_17_N_5_O_4_S (437.47): C, 57.66 (57.72); H, 4.38 (4.30); N, 16.01 (16.10).

### 3.27. General Procedure for Synthesis of 1-((4,6-Dimethyl-2-(oxo or thioxo)-1,2-dihydropyrimidin-5-yl)thio)-(7-methoxy or 7,11-dimethoxy)-6-methyl-3H-furo[3,2-g]pyrimido[1,6-a]quinazolin-3-one (***11a***–***d***)

A mixture of **9a** (4.11 g, 0.01 mol) or **9b** (4.41 g, 0.01 mol), and urea (0.60 g, 0.01 mol) or thiourea (0. 76 g, 0.01 mol) was stirred under reflux in dioxane (40 mL) in the presence of catalytic amount of piperidine (1 mL) for 16–18 h. The reaction mixture was allowed to cool to room temperature, poured into water (100 mL), the deposited precipitate was filtered off, washed with ethanol (40 mL), dried and crystallized from proper solvent to afford (**11a**–**d**).

### 3.28. Synthesis of 1-((4,6-Dimethyl-2-oxo-1,2-dihydropyrimidin-5-yl)thio)-7-methoxy-6-methyl-3H-furo[3,2- g]pyrimido[1,6-a]quinazolin-3-one (***11a***)

The compound was obtained from the reaction of (**9a**) with urea, as yellowish crystals, crystallized from dioxane in 86% yield, m.p. > 350 °C. IR (ν, cm^−1^) KBr: 3380 (brs, NH), 3032 (CH-aryl), 2942 (CH-aliph), 1685, 1680 (2C=O, amide), 1635 (C=N). ^1^H-NMR (DMSO-*d*_6_, ppm) δ 2.11 (s, 3H, CH_3_), 2.28 (s, 3H, CH_3_), 2.37 (s, 3H, CH_3_), 3.94 (s, 3H, OCH_3_), 6.72 (d, 1H, *J* = 2.31 Hz, furan), 7.36 (s, 1H, pyrimidine), 7.63 (s, 1H, phenyl), 7.79 (d, 1H, *J* = 2.38 Hz, furan), 10.60 (brs, NH, D_2_O exchangeable); ^13^C-NMR (DMSO-*d*_6_) δ 20.6, 20.9, 23.5 (3C, 3CH_3_), 61.8 (1C, OCH_3_), 85.4 (1C, pyrimidine), 91.5 (1C, CH, phenyl), 97.7 (1C, CH, pyrimidine), 100.4, 105.7, 108.2, 143.1, 146.1, 153.6, 158.5, 160.4, 163.8, 164.7, 165.2, 166.8 (12C, Ar-C), 169.2, 172.5 (2C, 2C=O); MS (70 eV, %) *m/z* 435 (M^+^, 92%); Anal. Calc. (Found) for C_21_H_17_N_5_O_4_S (435.46): C, 57.92 (57.85); H, 3.94 (3.99); N, 16.08 (16.15).

### 3.29. Synthesis of 1-((4,6-Dimethyl-2-thioxo-1,2-dihydropyrimidin-5-yl)thio)-7-methoxy-6-methyl-3H-furo[3,2-g]pyrimido[1,6-a]quinazolin-3-one (***11b***)

The compound was obtained from the reaction of (**9a**) with thiourea, as yellow crystals, crystallized from dimethylformamide in 82% yield, m.p. > 350 °C. IR (ν, cm^−1^) KBr: 3385 (brs, NH), 3035 (CH-aryl), 2944 (CH-aliph), 1681 (C=O, amide), 1632 (C=N). ^1^H-NMR (DMSO-*d*_6_, ppm) δ 2.10 (s, 3H, CH_3_), 2.27 (s, 3H, CH_3_), 2.36 (s, 3H, CH_3_), 3.91 (s, 3H, OCH_3_), 6.70 (d, 1H, *J* = 2.30 Hz, furan), 7.34 (s, 1H, pyrimidine), 7.61 (s, 1H, phenyl), 7.78 (d, 1H, *J* = 2.36 Hz, furan), 12.30 (brs, NH, D_2_O exchangeable); ^13^C-NMR (DMSO-*d*_6_) δ 21.4, 21.8, 23.7 (3C, 3CH_3_), 61.7 (1C, OCH_3_), 84.6 (1C, pyrimidine), 91.8 (1C, CH, phenyl), 98.2 (1C, CH, pyrimidine), 100.2, 105.8, 108.1, 143.7, 146.3, 153.8, 158.9, 160.5, 163.9, 164.2, 165.8, 169.6 (12C, Ar-C), 170.1 (1C, C=O), 177.8 (1C, C=S); MS (70 eV, %) *m/z* 451 (M^+^, 90%); Anal. Calc. (Found) for C_21_H_17_N_5_O_3_S_2_ (451.52): C, 55.86 (55.78); H, 3.80 (3.88); N, 15.51 (15.60).

### 3.30. Synthesis of 1-((4,6-Dimethyl-2-oxo-1,2-dihydropyrimidin-5-yl)thio)-7,11-dimethoxy-6-methyl-3H-furo[3,2-g]pyrimido[1,6-a]quinazolin-3-one (***11c***)

The compound was obtained from the reaction of (**9b**) with urea, as yellow crystals, crystallized from methanol in 80% yield, m.p. > 350 °C. IR (ν, cm^−1^) KBr: 3377 (brs, NH), 3039 (CH-aryl), 2937 (CH-aliph), 1687, 1683 (2C=O, amide), 1638 (C=N). ^1^H-NMR (DMSO-*d*_6_, ppm) δ 2.09 (s, 3H, CH_3_), 2.27 (s, 3H, CH_3_), 2.33 (s, 3H, CH_3_), 3.99 (s, 6H, 2OCH_3_), 6.75 (d, 1H, *J* = 2.32 Hz, furan), 7.38 (s, 1H, pyrimidine), 7.76 (d, 1H, *J* = 2.37 Hz, furan), 10.74 (brs, NH, D_2_O exchangeable); ^13^C-NMR (DMSO-*d*_6_) δ 20.5, 20.7, 23.7 (3C, 3CH_3_), 61.9 (2C, 2OCH_3_), 87.1(1C, pyrimidine), 98.9 (1C, CH, pyrimidine), 100.2, 105.4, 108.8, 122.3, 130.5, 146.1, 146.9, 152.1, 158.2, 164.1, 164.5, 166.2, 168.5 (13C, Ar-C), 169.8, 171.4 (2C, 2C=O); MS (70 eV, %) *m/z* 465 (M^+^, 88%); Anal. Calc. (Found) for C_22_H_19_N_5_O_5_S (465.48): C, 56.77 (56.70); H, 4.11 (4.05); N, 15.05 (15.14).

### 3.31. Synthesis of 1-((4, 6-Dimethyl-2-thioxo-1,2-dihydropyrimidin-5-yl)thio)-7,11-dimethoxy-6-methyl-3H-furo[3,2-g]pyrimido[1,6-a]quinazolin-3-one (***11d***)

The compound was obtained from the reaction of (**9b**) with thiourea, as yellowish crystals, crystallized from dioxane in 80% yield, m.p. > 350 °C. IR (ν, cm^−1^) KBr: 3388 (brs, NH), 3034 (CH-aryl), 2945 (CH-aliph), 1683 (C=O, amide), 1630 (C=N). ^1^H-NMR (DMSO-*d*_6_, ppm) δ 2.13 (s, 3H, CH_3_), 2.25 (s, 3H, CH_3_), 2.32 (s, 3H, CH_3_), 4.01 (s, 6H, 2OCH_3_), 6.73 (d, 1H, *J* = 2.34 Hz, furan), 7.36 (s, 1H, pyrimidine),7.80 (d, 1H, *J* = 2.38 Hz, furan), 12.50 (brs, NH, D_2_O exchangeable); ^13^C-NMR (DMSO-*d*_6_) δ 21.5, 21.9, 23.8 (3C, 3CH_3_), 62.01 (2C, 2OCH_3_), 86.1 (1C, pyrimidine), 99.3 (1C, CH, pyrimidine), 100.4, 105.2, 108.7, 122.7, 130.4, 146.3, 146.7, 151.9, 159.1, 164.3, 164.5, 166.9, 168.2 (13C, Ar-C), 169.8(1C, C=O), 178.5 (1C, C=S); MS (70 eV, %) *m/z* 481 (M^+^, 86%); Anal. Calc. (Found) for C_22_H_19_N_5_O_4_S_2_ (481.54): C, 54.87 (54.95); H, 3.98 (3.90); N, 14.54 (14.47).

### 3.32. General Procedure for Synthesis of (7-Methoxy or 7,11-dimethoxy)-6-methyl-1-(methylthio)-3H-furo[3,2-g]pyrimido[1,6-a]quinazolin-3-one (***12a***,***b***)

To a warmed ethanolic KOH solution (prepared by dissolving 10 mmol of KOH in 50 mL ethanol) was added each of **5a** (3.13 g, 0.01 mol) or **5b** (3.43 g, 0.01 mol), the heating was continued for 30 min and the mixture was allowed to cool to room temperature, and methyl iodide (0.62 mL, 0.01 mol) was added. The mixture was stirred under reflux for 5–7 h, then cool to room temperature, poured into cold water (100 mL). The solid product precipitated was filtered off, washed with water; the product was dried and crystallized from the proper solvent to produce **12a** and **12b**, respectively.

### 3.33. Synthesis of 7-Methoxy-6-methyl-1-(methylthio)-3H-furo[3,2-g]pyrimido[1,6-a]quinazolin-3-one (***12a***)

The compound was obtained from the reaction of (**5a**) with methyl iodide, as yellow crystals, crystallized from methanol in 90% yield, m.p. 329 °C. IR (ν, cm^−1^) KBr: 3035 (CH-aryl), 2962 (CH-aliph), 1685 (CO, amide), 1630 (C=N). ^1^H-NMR (DMSO-*d*_6_, ppm) δ 2.30 (s, 3H, CH_3_), 2.80 (s, 3H, SCH_3_), 3.90 (s, 3H, OCH_3_), 6.73 (d, 1H, *J* = 2.31 Hz, furan), 7.34 (s, 1H, pyrimidine), 7.64 (s, 1H, phenyl), 7.78 (d, 1H, *J* = 2.35 Hz, furan); ^13^C-NMR (DMSO-*d*_6_) δ 18.9 (1C, SCH_3_), 23.8 (1C, CH_3_), 61.4 (1C, OCH_3_), 92.7 (1C, CH, phenyl), 98.9 (1C, CH, pyrimidine), 100.3, 105.2, 108.5, 145.2, 146.4, 153.7, 158.3, 160.9, 164.6, 167.8 (10C, Ar-C), 169.1 (1C, C=O); MS (70 eV, %) *m/z* 327 (M^+^, 100%); Anal. Calc. (Found) for C_16_H_13_N_3_O_3_S (327.36): C, 58.71 (58.78); H, 4.00 (4.10); N, 12.84 (12.90).

### 3.34. Synthesis of 7,11-Dimethoxy-6-methyl-1-(methylthio)-3H-furo[3,2-g]pyrimido[1,6-a]quinazolin-3-one (***12b***)

The compound was obtained from the reaction of (**5b**) with methyl iodide, as yellowish crystals, crystallized from ethanol in 86% yield, m.p. 311 °C. IR (ν, cm^−1^) KBr: 3038 (CH-aryl), 2965 (CH-aliph), 1683 (CO, amide), 1633 (C=N). ^1^H-NMR (DMSO-*d*_6_, ppm) δ 2.35 (s, 3H, CH_3_), 2.88 (s, 3H, SCH_3_), 3.97 (s, 6H, 2OCH_3_), 6.78 (d, 1H, *J*=2.32 Hz, furan), 7.38 (s, 1H, pyrimidine), 7.80 (d, 1H, *J* = 2.34 Hz, furan); ^13^C-NMR (DMSO-*d*_6_) δ 19.2 (1C, SCH_3_), 23.6 (1C, CH_3_), 61.8 (2C, 2OCH_3_), 99.4 (1C, CH, pyrimidine), 100.1, 105.7, 108.2, 122.7, 130.5, 146.1, 146.8, 151.5, 159.7, 164.4, 168.2 (11C, Ar-C), 169.7 (1C, C=O); MS (70 eV, %) *m/z* 357 (M^+^, 100%); Anal. Calc. (Found) for C_17_H_15_N_3_O_4_S (357.38): C, 57.13 (57.22); H, 4.23 (4.28); N, 11.76 (11.66).

### 3.35. General Procedure for Synthesis of (7-Methoxy or 7,11-dimethoxy)-6-methyl-1-(methylsulfonyl)-3H- furo[3,2-g]pyrimido[1,6-a]quinazolin-3-one (***13a***,***b***)

A mixture of **12a** (3.27 g, 0.01 mol) or **12b** (3.57 g, 0.01 mol), and excess amount of hydrogen peroxide (5 mL) in acetic acid (40 mL) was heated gently with stirring for 12–14 h with (TLC). The reaction mixture was allowed to cool to 0 °C. The deposited precipitate was filtered off, and crystallized from the proper solvent to afford **13a** and **13b**, respectively.

### 3.36. Synthesis of 7-Methoxy-6-methyl-1-(methylsulfonyl)-3H-furo[3,2-g]pyrimido[1,6-a]quinazolin-3-one (***13a***)

The compound was obtained from the reaction of (**12a**) with hydrogen peroxide, as yellowish crystals, crystallized from benzene in 75% yield, m.p. 241 °C. IR (ν, cm^−1^) KBr: 3032 (CH-aryl), 2920 (CH-aliph), 1687 (CO, amide), 1632 (C=N), 1162, 1340 (SO_2_). ^1^H-NMR (DMSO-*d*_6_, ppm) δ 2.34 (s, 3H, CH_3_), 2.95 (s, 3H, SO_2_CH_3_), 3.92 (s, 3H, OCH_3_), 6.76 (d, 1H, *J* = 2.36 Hz, furan), 7.37 (s, 1H, pyrimidine), 7.61 (s, 1H, phenyl), 7.81 (d, 1H, *J* = 2.32 Hz, furan); ^13^C-NMR (DMSO-*d*_6_) δ 23.5 (1C, CH_3_), 35.6 (1C, SO_2_CH_3_), 61.3 (1C, OCH_3_), 93.5 (1C, CH, phenyl), 99.4 (1C, CH, pyrimidine), 100.2, 105.4, 108.7, 140.3, 145.5, 146.2, 153.9, 160.8, 164.4, 168.1 (10C, Ar-C), 169.5 (1C, C=O); MS (70 eV, %) *m/z* 359 (M^+^, 84%); Anal. Calc. (Found) for C_16_H_13_N_3_O_5_S (359.36): C, 53.48 (53.40); H, 3.65 (3.57); N, 11.69 (11.61).

### 3.37. Synthesis of 7,11-Dimethoxy-6-methyl-1-(methylsulfonyl)-3H-furo[3,2-g]pyrimido[1,6-a]quinazolin- 3-one (***13b***)

The compound was obtained from the reaction of (**12b**) with hydrogen peroxide, as yellow crystals, crystallized from toluene in 75% yield, m.p. 221 °C. IR (ν, cm^−1^) KBr: 3029 (CH-aryl), 2918 (CH-aliph), 1684 (CO, amide), 1628 (C=N), 1160, 1342(SO_2_). ^1^H-NMR (DMSO-*d*_6_, ppm) δ 2.29 (s, 3H, CH_3_), 2.97 (s, 3H, SO_2_CH_3_), 4.01 (s, 6H, 2OCH_3_), 6.80 (d, 1H, *J* = 2.30 Hz, furan), 7.31 (s, 1H, pyrimidine), 7.75 (d, 1H, *J* = 2.33 Hz, furan); ^13^C-NMR (DMSO-*d*_6_) δ 23.3 (1C, CH_3_), 35.8 (1C, SO_2_CH_3_), 61.9 (2C, 2OCH_3_), 99.8 (1C, CH, pyrimidine), 100.4, 105.7, 108.9, 122.4, 130.1, 140.6, 146.3, 146.8, 151.5, 164.7, 168.5(11C, Ar-C), 169.5 (1C, C=O); MS (70 eV, %) *m/z* 389 (M^+^, 80%); Anal. Calc. (Found) for C_17_H_15_N_3_ O_6_S (389.38): C, 52.44 (52.50); H, 3.88 (3.80); N, 10.79 (10.71).

### 3.38. General Procedure for Synthesis of (7-Methoxy or 7,11-dimethoxy)-6-methyl-1-((piperazin-1-yl) or morpholino)-3H-furo[3,2-g]pyrimido[1,6-a]quinazolin-3-one (***14a***–***d***)

In a warm solution of **12a** (3.27 g, 0.01 mol) or **12b** (3.57 g, 0.01 mol), in methanol (50 mL) was added the freshly distilled 2nd amine, namely piperazine (0.95 mL, 0.01 mol) or morpholine (0.86 mL, 0.01 mol). The reaction mixture was stirred under reflux for 7–9 h, then allowed to cool to 0 °C for 14 h and the solid obtained was filtered, washed with water (100 mL) dried and recrystallized from appropriate solvent to produce (**14a**–**d**).

### 3.39. Synthesis of 7-Methoxy-6-methyl-1-(piperazin-1-yl)-3H-furo[3,2-g]pyrimido[1,6-a]quinazolin-3-one (***14a***)

The compound was obtained from the reaction of (**12a**) with piperazine, as pale yellow crystals, crystallized from dioxane in 79% yield, m.p. 202 °C. IR (ν, cm^−1^) KBr: 3390 (br, NH), 3044 (CH-aryl), 2940 (CH-aliph), 1687 (CO, amide), 1620 (C=N). ^1^H-NMR (DMSO-*d*_6_, ppm) δ 2.29 (s, 3H, CH_3_), 2.82–2.91 (m, 8H, piperazine), 3.92 (s, 3H, OCH_3_), 6.78 (d, 1H, *J* = 2.36 Hz, furan), 7.38 (s, 1H, pyrimidine), 7.66 (s, 1H, phenyl), 7.81 (d, 1H, *J* = 2.37 Hz, furan), 10.30 (brs, NH, D_2_O exchangeable); ^13^C-NMR (DMSO-*d*_6_) δ 23.2 (1C, CH_3_), 48.3 (2C, 2CH_2_, piperazine), 52.5 (2C, 2CH_2_, piperazine), 61.7 (1C, OCH_3_), 91.8 (1C, CH, phenyl), 98.4 (1C, CH, pyrimidine), 100.1, 105.3, 108.2, 144.6, 146.1, 153.8, 154.9, 160.3, 164.4, 168.1(10C, Ar-C), 169.3 (1C, C=O); MS (70 eV, %) *m/z* 365 (M^+^, 83%); Anal. Calc. (Found) for C_19_H_19_N_5_O_3_ (365.39): C, 62.46 (62.55); H, 5.24 (5.18); N, 19.17 (19.24).

### 3.40. Synthesis of 7-Methoxy-6-methyl-1-morpholino-3H-furo[3,2-g]pyrimido[1,6-a]quinazolin-3-one (***14b***)

The compound was obtained from the reaction of (**12a**) with morpholine, as yellow crystals, crystallized from methanol in 75% yield, m.p. 171 °C. IR (ν, cm^−1^) KBr: 3040 (CH-aryl), 2936 (CH-aliph), 1685 (CO, amide), 1622 (C=N). ^1^H-NMR (DMSO-*d*_6_, ppm) δ 2.30 (s, 3H, CH_3_), 3.14–3.23 (m, 8H, morpholine), 3.90 (s, 3H, OCH_3_), 6.76 (d, 1H, *J* = 2.34 Hz, furan), 7.33 (s, 1H, pyrimidine), 7.64 (s, 1H, phenyl), 7.80 (d, 1H, *J* = 2.35 Hz, furan); ^13^C-NMR (DMSO-*d*_6_) δ 23.1 (1C, CH_3_), 51.5 (2C, 2CH_2_, morpholine), 55.8 (2C, 2CH_2_, morpholine), 61.9 (1C, OCH_3_), 91.5 (1C, CH, phenyl), 99.5 (1C, CH, pyrimidine), 100.7, 105.6, 108.4, 144.7, 146.3, 153.9, 155.2, 160.7, 164.8, 168.9 (10C, Ar-C), 169.8 (1C, C=O); MS (70 eV, %) *m/z* 366 (M^+^, 78%); Anal. Calc. (Found) for C_19_H_18_N_4_O_4_ (366.38): C, 62.29 (62.37); H, 4.95 (4.88); N, 15.29 (15.22).

### 3.41. Synthesis of 7,11-Dimethoxy-6-methyl-1-(piperazin-1-yl)-3H-furo[3,2-g]pyrimido[1,6-a]quinazolin-3-one (***14c***)

The compound was obtained from the reaction of (**12b**) with piperazine, as yellowish crystals, crystallized from ethanol in 74% yield, m.p. 184 °C. IR (ν, cm^−1^) KBr: 3395 (br, NH), 3048 (CH-aryl), 2944 (CH-aliph), 1689 (CO, amide), 1624 (C=N). ^1^H-NMR (DMSO-*d*_6_, ppm) δ 2.31 (s, 3H, CH_3_), 2.85–2.94 (m, 8H, piperazine), 3.97 (s, 6H, 2OCH_3_), 6.81 (d, 1H, *J* = 2.32 Hz, furan), 7.36 (s, 1H, pyrimidine), 7.84 (d, 1H, *J* = 2.30 Hz, furan), 10.35 (brs, NH, D_2_O exchangeable); ^13^C-NMR (DMSO-*d*_6_) δ 23.5 (1C, CH_3_), 48.6 (2C, 2CH_2_, piperazine), 52.8 (2C, 2CH_2_, piperazine), 61.8 (2C, 2OCH_3_), 99.6(1C, CH, pyrimidine), 100.5, 105.7, 108.9, 122.3, 130.2, 146.4, 146.5, 151.8, 154.1, 164.8, 168.5 (10C, Ar-C), 169.7(1C, C=O); MS (70 eV, %) *m/z* 395 (M^+^, 76%); Anal. Calc. (Found) for C_20_H_21_N_5_O_4_ (395.42): C, 60.75 (60.82); H, 5.35 (5.43); N, 17.71 (17.78).

### 3.42. Synthesis of 7,11-Dimethoxy-6-methyl-1-morpholino-3H-furo[3,2-g]pyrimido[1,6-a]quinazolin-3-one (***14d***)

The compound was obtained from the reaction of (**12b**) with morpholine, as yellowish crystals, crystallized from n-hexane in 72% yield, m.p. 156 °C. IR (ν, cm^−1^) KBr: 3039 (CH-aryl), 2934 (CH-aliph), 1683 (CO, amide), 1626 (C=N). ^1^H-NMR (DMSO-*d*_6_, ppm) δ 2.32 (s, 3H, CH_3_), 3.16–3.25 (m, 8H, morpholine), 3.97 (s, 6H, 2OCH_3_), 6.79 (d, 1H, *J* = 2.33 Hz, furan), 7.37 (s, 1H, pyrimidine), 7.82 (d, 1H, *J* = 2.37 Hz, furan); ^13^C-NMR (DMSO-*d*_6_) δ 23.7 (1C, CH_3_), 53.8 (2C, 2CH_2_, morpholine), 57.6 (2C, 2CH_2_, morpholine), 61.9 (2C, 2OCH_3_), 99.8 (1C, CH, pyrimidine), 100.4, 105.3, 108.7, 122.6, 130.1, 146.2, 146.8, 151.3, 154.2, 164.5, 168.6 (11C, Ar-C), 169.4 (1C, C=O); MS (70 eV, %) *m/z* 396 (M^+^, 74%); Anal. Calc. (Found) for C_20_H_20_N_4_O_5_ (396.40): C, 60.60 (60.68); H, 5.09 (5.16); N, 14.13 (14.20).

### 3.43. Biological Screening

The antimicrobial activity of the newly prepared compounds was tested in vitro against Gram-positive bacteria *Staphylococcus aureus* (ATCC^®^6538™), *Streptococcus pyogenes* (ATCC^®^19615™), Gram-negative bacteria *Escherichia coli* (ATCC^®^25922™), *Klebsiella pneumoniae* (ATCC^®^ 10031™) and the fungi *Aspergillus niger* (ATCC^®^ 16888™), *Alternaria alternate, Curvularia lunata* and *Candida albicans* (ATCC^®^10231™). The newly prepared compounds were dissolved in dimethyl sulfoxide (DMSO) and tested for their antimicrobial activity by the agar disk diffusion technique. Cefotaxime sodium and nystatin [34,35] were used as the standard drugs for antibacterial and antifungal assays, respectively. A solution of 100 μg mL^−1^ of the tested compound was practical and microplate-wells, 1 cm in diameter, were used. Zones of inhibition were measured with calipers or automated scanners and were paralleled with those of the standards. Cefotaxime sodium (0.15 μmol mL^−1^) and nystatin (0.037 μmol mL^−1^) were used as the standard drugs for antibacterial and antifungal activity, respectively. Compound-impregnated disks were placed on an agar plate containing a standard suspension of microorganisms. The plate was incubated for 24 h at 37 °C. For the assessment of the minimum inhibitory concentration (MIC) by the serial plate dilution way [34,35], 5 mg of each tested compound was dissolved in 1 mL of DMSO separately to prepare stock solutions. Serial dilutions were prepared from each stock solution. The plates were incubated at 37 °C for 24 h. MIC is defined as the lowest concentration (μmol mL^−1^) of the tested compound that results in no visible growth on the plates. DMSO was used as the solvent control to ensure that the solvent had no effect on bacterial growth. The results are shown in Table 1 and Table 2.

## 4. Conclusions

In this work, we synthesized novel heterocyclic compounds with potent antimicrobial activity starting from furochromones (visnagenone **2a** or khellinone **2b**). New derivatives were prepared in good yields such as; furopyrimido quinazolinones, furothiazolo pyrimido quinazolinones, substituted-benzyliden-furothiazolo pyrimido quinazolinones, pyrazolo furopyrimido quinazolin-ones, oxo or thioxo-pyrimidin-furopyrimido quinazolinones and methylthio, methylsulfonyl, piperazino or morpholino-furopyrimido quinazolinones. Some new prepared compounds such as substituted-benzylidene-furothiazolo pyrimido quinazolinones **8a**–**f** showed higher activity against the tested microorganisms (bacteria and fungi).

## Figures and Tables

**Table 1:** A minimum inhibitory concentration of the compounds against bacteria.

MIC (µmol mL^−1^)				
Compounds	Microorganisms			
	Gram-Positive Bacteria		Gram-Negative Bacteria	
	*Staphylococcus Aureus*	*Streptococcus Pyogenes*	*E. coli*	*Klebsiella Pneumoniae*
**1a**	34	33	31	30
**1b**	33	32	30	29
**2a**	31	30	29	28
**2b**	30	29	28	27
**3**	37	35	34	33
**4a**	29	28	27	26
**4b**	28	27	26	25
**5a**	27	26	25	24
**5b**	26	25	24	23
**6a**	23	22	21	20
**6b**	22	21	20	19
**7a**	17	16	15	14
**7b**	16	15	14	13
**8a**	8	7	7	5
**8b**	5	4	3	2
**8c**	7	6	5	4
**8d**	7	7	6	4
**8e**	4	3	2	1
**8f**	6	5	4	3
**9a**	19	18	17	16
**9b**	18	17	16	15
**10a**	12	11	10	10
**10b**	11	11	10	9
**11a**	10	10	9	8
**11b**	9	8	7	7
**11c**	10	9	8	7
**11d**	8	8	7	6
**12a**	25	24	23	22
**12b**	24	23	22	21
**13a**	21	20	19	18
**13b**	20	19	18	17
**14a**	14	13	12	11
**14b**	15	14	14	12
**14c**	13	12	11	10
**14d**	14	14	13	12
Cefotaxime sodium	5	4	3	2
Negative control	NI	NI	NI	NI

DMSO was used as the negative control and as the solvent for test compounds and the reference drug. NI–No inhibition.

**Table 2:** A minimum inhibitory concentration of the compounds against fungi.

MIC (µmol mL^−1^)				
Compounds	Microorganisms			
	*Aspergillus Niger*	*Alternaria Alternata*	*Curvularia Lunata*	*Candida Albicans*
**1a**	39	38	37	36
**1b**	38	36	34	33
**2a**	35	34	33	32
**2b**	33	32	31	30
**3**	42	40	39	38
**4a**	31	30	29	28
**4b**	30	29	27	26
**5a**	29	28	26	25
**5b**	29	27	26	25
**6a**	26	24	23	22
**6b**	25	23	22	21
**7a**	20	18	17	16
**7b**	19	17	16	15
**8a**	9	8	8	4
**8b**	6	5	4	3
**8c**	8	7	6	5
**8d**	9	8	7	5
**8e**	5	4	3	2
**8f**	7	6	5	4
**9a**	22	20	19	18
**9b**	21	19	18	17
**10a**	14	13	12	11
**10b**	13	12	11	10
**11a**	12	11	10	9
**11b**	10	9	8	8
**11c**	11	10	9	8
**11d**	9	9	8	7
**12a**	28	26	25	24
**12b**	27	25	24	23
**13a**	24	22	21	20
**13b**	23	21	20	19
**14a**	16	15	14	13
**14b**	18	16	15	15
**14c**	15	14	13	12
**14d**	17	16	15	14
Nystatin	4	3	2	2
Negative control	NI	NI	NI	NI

DMSO was used as the negative control and as the solvent for test compounds and the reference drug. NI–No inhibition.
